# The Role of Innate Immunity in Pulmonary Infections

**DOI:** 10.1155/2021/6646071

**Published:** 2021-01-22

**Authors:** Huihui Zhang, Fang He, Pan Li, Philip R. Hardwidge, Nengzhang Li, Yuanyi Peng

**Affiliations:** ^1^College of Animal Medicine, Southwest University, Chongqing, China; ^2^College of Veterinary Medicine, Kansas State University, Manhattan, KS, USA

## Abstract

Innate immunity forms a protective line of defense in the early stages of pulmonary infection. The primary cellular players of the innate immunity against respiratory infections are alveolar macrophages (AMs), dendritic cells (DCs), neutrophils, natural killer (NK) cells, and innate lymphoid cells (ILCs). They recognize conserved structures of microorganisms through membrane-bound and intracellular receptors to initiate appropriate responses. In this review, we focus on the prominent roles of innate immune cells and summarize transmembrane and cytosolic pattern recognition receptor (PRR) signaling recognition mechanisms during pulmonary microbial infections. Understanding the mechanisms of PRR signal recognition during pulmonary pathogen infections will help us to understand pulmonary immunopathology and lay a foundation for the development of effective therapies to treat and/or prevent pulmonary infections.

## 1. Introduction

Lung tissue is continuously exposed to various pathogenic microorganisms in the environment, leading to lung infections or lung diseases. The innate immune system is the first line of defense against pathogens and includes a range of immune cells and related mechanisms that nonspecifically recognize and resist infections [1–4]. The main pathogens of lung infection include *Streptococcus pneumoniae*, *Staphylococcus aureus*, *Legionella pneumophila*, *Chlamydia pneumoniae*, *Klebsiella pneumoniae*, and *Pseudomonas aeruginosa* [4–7]. The rapid identification of nonself-exogenous pathogens and their effective elimination with a complex set of defense mechanisms is a testament to the efficiency of the innate immune cells within the airways and lungs. Innate immune cells include alveolar macrophages (AMs), neutrophils, dendritic cells (DCs), natural killer cells (NK), and large mononuclear cells, which can recognize pathogen-associated molecular patterns (PAMPs) such as components of bacteria via their pattern-recognition receptors (PRRs) [8, 9].

Successful pathogenic identification and appropriate responses are essential for effective pulmonary host defense [10]. One of the primary mechanisms for bacterial growth containment at the site of infection and consequently for minimizing bacterial dissemination is the PRR-mediated innate immune responses. PRRs, such as Toll-like receptor (TLRs), Nod-like receptors (NLRs) [11], and retinoic acid inducible gene I- (RIG-I-) like receptors (RLRs) [12], are involved in innate immune responses and/or apoptosis [13]. Therefore, PRR-mediated signaling pathways play a significant role in the synergistic inflammatory responses and the balance of tissue homeostasis.

TLRs are important for the pathogen recognition and host immune response initiation. Human and mice contain 10 (TLR1-10) and 12 (TLR1-9, 11-13) TLRs, respectively [14]. TLR1, TLR2, TLR4, TLR5, TLR6, and probably TLR12 of mice and TLR10 of humans are located on the cell surface [14, 15]. TLR3, TLR7, TLR8, and TLR9 localize in intracellular vesicles such as endoplasmic reticulum (ER), endosome, lysosome, and endolysosome [14, 15]. Recent studies have shown that TLR11 is expressed both extracellularly and intracellularly in human cells [15, 16]. TLR13 is also expressed in intracellular vesicles [15]. Plasma membrane-bound TLRs (TLR2/1, TLR2/6, TLR4, and TLR5) and endosome membrane-bound TLRs (TLR2/1, and TLR9) are mainly involved in the recognition of lung bacteria [17, 18], while TLR3, TLR7, and TLR8 recognize nucleic acids of viruses during pulmonary infections [19].

NLRs are expressed in the nucleus and cytoplasm [20]. There are more than 20 receptors in the NLR family, which have three domains: the N-terminal effector domain, the central NOD domain, and the leucine-rich repeat C-terminal domain [20]. N-terminal functional domains are divided into four groups: transactivator activation domain, baculovirus inhibitor of apoptosis (BIR), caspase recruitment domain (CARD), and pyrin domain (PYD) [12, 21]. NLRPs (nucleotide-binding oligomerization domain-like receptors proteins) are key molecules in the innate immune response to pulmonary infection. The NLRP of NLRs are made up of 14 proteins, of which NLRP1, NLRP3, NLRP6, NLRP7, and NLRP12 form inflammasomes, consisting of NLRP, ASC, and procaspase-1 [11, 20]. Inflammasomes formed by these NLRs regulate cytokine IL-1 family and pyrolytic cell death, nuclear factor kappa-light-chain-enhancer of activated B cell- (NF-*κ*B-) dependent inflammatory mediators, autophagy, or reactive oxygen species (ROS) production [11, 20]. In addition, unlike other NLR members, the N-terminus of NLRCs contain a special CARD (apoptosis-associated speck-like protein (ASC)), which is also known as the death domain (DD) folding zone [22]. Different NLRs can detect different lung pathogens. Several inflammasomes are activated as part of the host innate immune response during different bacterial infections.

This review discusses recent advances on the function of innate immune cells in lung bacterial infections, and highlights the mechanisms used by pathogens to modulate or interfere with PRR relevant signaling in the pulmonary antibacterial responses for bacterial pathogens.

## 2. Innate Immune Cells in Pulmonary Infections

### 2.1. Alveolar Macrophages in Pulmonary Infections

There are three types of permanent tissue macrophages in the lung, including AMs, interstitial macrophages, and bronchial macrophages [23]. These cells mediate opsonophagocytosis and nonopsonophagocytosis of inhaled or exhaled pathogens [24]. AMs play a central role in maintaining environmental stability and inducing effective defense mechanisms [25]. Pathogens such as *L. pneumophila*, *Actinobacillus pleuropneumoniae*, and *Mycobacterium tuberculosis* invade the lung and activate AMs to produce proinflammatory cytokines and chemokines, such as interleukin-1*α*/*β* (IL-1*α*/*β*), IL-6, tumor necrosis factor-*α* (TNF-*α*), interferon-*α*/*β* (IFN-*α*/*β*), and CXC chemokine ligand (CXCL2) [26–33]. Under the pathological conditions of inflammation, injury, and infection, the subgroup of AMs is different in the lung, namely, tissue-resident AMs (TR-AMs) and monocyte-derived AMs (Mo-AMs) [34, 35]. Since mice infected with viruses consume a large amount of TR-AMs and are accompanied by a large number of circulating blood mononuclear cells into the lungs, Mo-AMs become increasingly indistinguishable from TR-AMs as the lung returns to homeostasis [36, 37]. It is not well understood whether the functions of macrophages with different origins and locations are the same or whether they have the ability to transform. Each macrophage lineage arrives in the lung at a different time, destined to become a specific type of macrophage with a unique microanatomical niche and renewal mechanism [38]. Recent studies have presented compelling evidence that Mo-AM apolipoprotein E is beneficial to the resolution of lung fibrosis, supporting the notion that Mo-AMs may have distinct functions in different phases of lung fibrogenesis [39]. Therefore, exploring the number and function of colonized AMs and AMs differentiated by circulating blood monocytes, lung mesenchymal macrophages, and their relationship with each other is of significance for studying inflammatory mechanisms caused by pathogenic bacteria.

### 2.2. Neutrophils in Pulmonary Infections

Neutrophils are involved in the removal of exogenous and endogenous cellular debris and play an essential role in the pathogenesis of many respiratory infections. Acute lung inflammation, triggered by neutrophils, can be viewed as pathogenic, because their activation promotes further damage in the early phases of the inflammatory response [40]. Once specific receptors (such as a toll-like receptor) recognize antigen, releasing a cascade of mediators and leading to a chemotactic signal, neutrophils are recruited into the lung interstitium followed by a transepithelial migration into the alveolar space [41, 42]. For example, *P. aeruginosa*, LPS, and *β*-glucans promote the recruitment of circulating neutrophils, in which proinflammatory cytokine production (TNF-*α*, IFN-*γ*, and IL-8) and inflammatory chemokines (the chemokine (C-C motif) ligand (CCL) 2 and CCL7) act synergistically to participate in lung inflammation [43–47]. These migration steps are regulated differently by the interaction of neutrophilic adhesion molecules (including CD11a, CD11b, CD44, CD162, CD29, CD54, CD47, CD31, and CD172a) [48]. These neutrophils phagocytose bacteria with the release of proteases (such as neutrophil elastase), cytotoxic molecules, and reactive oxygen species, enhancing inflammation and resulting in host damage [47, 49].

However, this neutrophilic infiltration also plays a role in the late phases of damaged areas for tissue regeneration [41, 42]. There are some molecular mechanisms by which neutrophils could orchestrate lung repair [50–53]. The most common mechanisms include neutrophil extracellular traps (NETs). Some of the matrix metalloproteinase (including MMP-2, 8, 9) and proresolving lipid mediators (including lipoxin A4, resolvins, and protectins) released by neutrophils directly contribute to tissue remodeling and repair [40, 50, 54, 55]. Neutrophil transmigration promotes tissue remodeling and repair by Wnt/ß-catenin-dependent pathways with the release of cysteine-rich angiogenic inducer 61 (Cyr61) [56–58]. In addition, neutrophils may intercellularly transfer *miR-223* to epithelial cells to dampen acute lung injury through repression of poly (adenosine diphosphate-ribose) polymerase–1 (PARP-1) [59]. CXCL1 orchestrates neutrophil homeostasis in *Klebsiella pneumoniae*- and *S. pneumoniae*-induced lung inflammation and sepsis [44, 60]. Overall, these mediators stimulate apoptotic neutrophils and block neutrophil recruitment at late stages of the acute response through NETs capturing released chemokines (e.g., CCL5, CXCL12, and CXCL4) [42, 61].

### 2.3. Dendritic Cells in Pulmonary Infections

DCs, as antigen-presenting cells that connect the innate and adaptive immune systems, ensure an effective immune response during infection. Importantly, there are functional differences between the different subsets of lung DCs. Subpopulations of DC are classified into CD103^+^ CD11b^−^ conventional DCs (cDC1), CD103^−^ CD11b^+^cDCs (cDC2), and plasmacytoid DCs (pDC) in the lungs [62–65]. cDCs, as proinflammatory initiators and mediators, shifting balance between Th1 and Th2 responses and crosstalk with neutrophils, may be two underlying mechanisms during bacterial pathogen-induced acute lung inflammation and injury [66]. Pulmonary CD103^+^ DCs (CD103^+^ PDCs) is beneficial for T helper 2 cell (Th2) and Th17 immunity, while CD103^+^cDCs is associated with the induction of Th1, cytotoxic T lymphocyte (CTL) responses, and regulatory T cells [67–70]. Following *Klebsiella pneumonia* infection, the detection of increased respiratory CD103^+^ PDC numbers enhanced antigen-specific CD4^+^ T cell responses, which may indicate possible novel PDC functions with respect to lung repair and regeneration [71]. Using transgenic mice enabling the inducible depletion of CD103^+^ DCs found that DC subset contributes to the control of mycobacterial burden, which was associated with consistently reduced levels of total and activated CD4^+^ and CD8^+^ T cells and Th1-related cytokines (IFN-*γ* and TNF-*α*) [72]. *Pasteurella multocida* triggers the maturation of DCs and IL-12 production, a cytokine known to induce differentiation of native T cells into interferon-*γ*- (IFN-*γ*-) producing Th1 cells, which are resistant to infection [73, 74]. These findings suggest that bacteria target the high plasticity of T cell subtypes to enhance their pathogenicity and may gain advantages in survival and reproduction. Clearly, more work is required to address (i) the mechanism of DCs in manipulating native T cell differentiation and (ii) how to prolong neutrophil survival during bacterial pathogen infection.

### 2.4. Natural Killer Cells in Pulmonary Infections

NK cells constitute the first line of defense against pathogenic microorganisms. There is increasing evidence that under steady-state conditions, the frequency of NK cells in the total lymphocyte population of the lungs is high, and lung NK cells have a more mature phenotype, suggesting that quick and effective NK-mediated immune responses are critical for eliminating pathogens and maintaining homeostasis in the lung [75–77]. Previous studies have indicated that IFN-*γ*, IL-21, and IL-22 produced by NK cells enhance the immune response through increasing IL-1*β*, IL-18, and MIP-1*β* production and reducing IL-10 expression of monocytes in response to an intracellular pathogen in the lungs [78–82]. The mechanism by which NK cells protect against bacterial infection has not been extensively characterized but may include the production of cytokines such as tumor necrosis factor (TNF) and IFN-*γ*, the production of chemokines to recruit additional leukocytes, interactions with macrophages to regulate bacterial clearance, and direct bacterial killing [78, 80, 83].

Innate lymphoid cells (ILCs, e.g., NK cells, ILC1s, ILC2s, ILC3s, and lymphoid tissue-inducer cells) play important roles in the protective immunity of the pulmonary infections [84–88]. T-bet^+^ ILC1s produce IFN-*γ*. GATA3^+^ ILC2s secrete IL-5, IL-9, and IL-13. Ror*γ*t^+^ILC3s produce IL-22 and IL-17 [86, 89, 90]. Upon detection of a signal from damaged epithelial cells, ILC2s release a large number of cytokines including IL-4, IL-5, IL-13, killer cell lectin-like receptor subfamily G member 1 (KLRG1), transforming growth factor-beta (TGF-*β*), and amphiregulin, which are also involved in lung tissue repair, immune response, and maintenance of tissue homeostasis [85, 88, 91–95]. Generally, the function of ILC2 is inhibited by ILC1 via IFN-*γ* production [85]. IL-22, the cytokine produced by NK cells and ROR*γ*t-expressing ILC3s, is involved in defense against rodent-adapted *Klebsiella pneumoniae* and *Streptococcus pneumoniae* [96, 97]. Thus, although other ILCs in the lungs are rare than NK cells, ILC1s and ILC3s facilitate perplex with NK cells [98, 99], so previous studies on the production of cytokine IL-22 and IFN-*γ* by lung NK cells may be affected by the effects of other ILCs. In addition, the role of lung ILC3 is still unclear in respiratory infection, but some of these effects have been demonstrated in maintenance of homeostasis, infection, and other mucosal barriers [100]. Therefore, further study is needed to explore the similarity and difference function of lung NK cells and other ILCs.

Recent studies provide some evidences that a prolonged alteration or enhancement in antimicrobial function of innate immune cells can itself contribute to protection from secondary infection [101]. This functional reprogramming of innate immune cells such as myeloid and NK cells, termed trained immunity [102]. From this point of view, we discuss the response of innate immunity to such as Bacille Calmette Guérin (BCG) and Pneumococcal Polysaccharide Vaccine (PPV). For example, Bacille Calmette Guérin (BCG) vaccine utilized an attenuated strain of *Mycobacterium bovis* protect chronic infection disease threats such as tuberculosis (TB) through activating NOD2 [103, 104]. Recent studies demonstrated that respiratory mucosal TB vaccination alters the airway innate immune landscape associated with airway macrophages prior to *M. tuberculosis* exposure and vaccine-trained airway macrophages enhance anti-TB innate immunity [105, 106]. In addition, BCG revaccination of adults with latent TB infection also induces long-lived BCG-reactive NK cell responses [107]. Previous works reveal great functional plasticity of the lungs of mice in defense against a bacterial pathogen through activation of innate immunity protects [108]. PPV is effective in preventing invasive pneumococcal infections in immunocompetent patients with indications for receiving the vaccine [109, 110]. Additionally, macrophage-mediated innate immunity activated through Pneumococcal conjugate vaccination (PCV) treatment intracellular killing of secondary *S. pneumoniae* [111]. It is important to investigate enhancement of the pulmonary innate antimicrobial defenses especially in the complex context of sequential infection or coinfection.

## 3. Innate Immune Recognition Mechanisms in Pulmonary Infections

Bacteria binds cell receptors, such as intercellular adhesion molecule 1 (ICAM-1), sialic acid, platelet activating factor (PAF), and glycine 6-phosphate receptor to recognize PRRs downstream signaling [112]. In this part, we will summarize the current knowledge of the multiple roles of TLRs in lung bacterial infections and highlight the mechanisms by which pathogens regulate or interfere with TLR signaling in the lungs. In order to clarify the complex NLR signaling network, we reviewed the roles of 7 different NLR family members (NOD1, NOD2, NLRC4, NLRC5, NLRP1, NLRP3, NLRP6, NLRP7, and NLRP12) involved in lung infections.

### 3.1. Toll-Like Receptors in Pulmonary Infections

#### 3.1.1. Plasma Membrane-Bound TLRs

**Figure 1:**
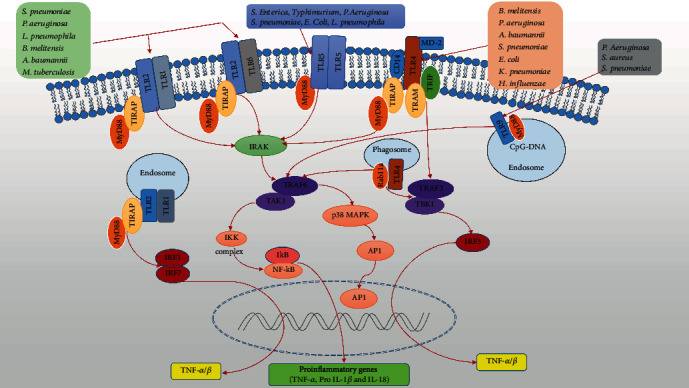
Overview of the Toll-like receptor-signaling pathway during pulmonary infections. Plasma membrane-bound TLRs (TLR1, 2, 4, 5, 6) and endosome membrane-bound TLRs (TLR9) recognize bacteria in the lungs. After bacterial recognition, TLR2 with TLR1 or TLR6 and TLR4 (in association with MD-2 and CD14) recruit TIRAP and MyD88, whereas TLR5 and TLR9 recruit MyD88. TLRs activate IRAK, followed by recruitment of TRAF6, eventually leading to activation of NF-*κ*B and MAPKs to the nucleus and the expression of inflammatory cytokines. However, in inflammatory monocytes infected with viruses, TLR2 is activated within the endosome, followed by induction of type I IFN via unique MyD88-dependent activation of IRF3 and IRF7. In addition, TLR4 also recruits the adaptor TRIF and activates TRAF3-TBK1-IRF3 axis to produce type I IFN. TLR: Toll-like receptor; MyD88: myeloid differential protein-88; TIRAP: Toll-IL-1R domain-containing adapter protein; IRAK: IL-1 receptor-associated kinase; TRAF6: tumor necrosis factor receptor-associated factor 6; NF-*κ*B: nuclear factor-*κ*B; MAPKs : mitogen-activated protein kinases; TRIF: TIR domain-containing adapter inducing IFN-*β*; IRF: IFN regulatory factor; TRAM: TRIF-related adapter molecule; ssRNA: single-stranded RNA; dsRNA: double-stranded RNA; *A. baumannii*: *Acinetobacter baumannii*; *E. coli*: *Escherichia coli*; *K. pneumoniae*: *Klebsiella pneumoniae*; *L. pneumophila*: *Legionella pneumophila*; *H. influenzae*: *Haemophilus influenzae*; *M. tuberculosis*: *Mycobacterium tuberculosis*; *P. aeruginosa*: *Pseudomonas aeruginosa*; *S. pneumoniae*: *Streptococcus pneumoniae*; *B. melitensis*: *Brucella melitensis*; *S. enterica*: *Salmonella enterica.*

*(1) TLR2-TLR1/TLR6*. TLR2 recognizes a variety of PAMPs, including lipoproteins, lipopeptides, *β*-glucans, glycoproteins, and zymosans, thereby recruiting TIR domain-containing adaptor protein (e.g., myeloid differentiation factor 88 (MyD88), Toll-IL-1R domain-containing adapter protein (TIRAP), TIR domain-containing adapter inducing IFN-*β* (TRIF), or TRIF-related adapter molecule (TRAM)) [14, 113]. TLR2 signaling is essential for bacterial clearance and survival in the lung of mice infected with *S. pneumoniae*, *P. Aeruginosa*, *L. pneumophila*, *Brucella melitensis*, *Acinetobacter baumannii*, *Mycoplasma*, *M. tuberculosis*, and *S. aureus*, indicating the importance of the TLR2 pathway for protecting the host [13, 114–124] (Figure 1). In most cases, TLR2 forms heterodimers with TLR1 and/or TLR6 [122]. After bacterial recognition, heterodimers of TLR1-TLR2 and TLR2-TLR6 on the surface of macrophages and cDCs activate NF-*κ*B through the recruitment of TIRAP and MyD88 to induce the expression of inflammatory cytokines [125]. Synthetic bacterial lipopeptides are recognized from within endosomes by TLR2/1 [126, 127]. TLR2/1 is activated within the endosome and induces type I IFN via unique MyD88-dependent activation of IFN-regulatory factor 3 (IRF3) and IRF7 [127–129] (Figure 1).

*(2) TLR4*. Plasma membrane-bound TLR4 recognizes a variety of gram-negative bacterial LPS, including *B.melitensis*, *P. aeruginosa*, *A. baumannii*, *S. pneumoniae*, *E. coli*, *K. pneumoniae*, and *Haemophilus influenzae* [130–133]. LPS binds to CD14 (Cluster of Differentiation), thereby promoting the transfer of LPS to the TLR4/MD-2 complex, activating p38, extracellular signal-regulated kinases (ERK), and c-Jun N-terminal kinases (JNKs). Macrophages and primordial human alveolar type II (ATII) cells release large amounts of TNF-*α*, IL-8, and monocyte chemoattractant protein 1 (MCP-1) [134]. *M. pneumoniae* activates mitogen-activated protein kinases (MAPKs) by recognizing TLR2 and TLR4 and activates NF-*κ*B via MyD88 to produce inflammatory factors such as pro-IL-1*β* [135]. After bacterial recognition, TLR4 initially recruits TIRAP and MyD88 and then activates NF-*κ*B and p38 MAPK via interleukin-1 receptor-associated kinases (IRAKs), TNF receptor-associated factor 6 (TRAF6), and TGF beta-activated kinase 1 (TAK1) complexes [122, 136–141] (Figure 1). Furthermore, TLR4 is endocytosed to the Rab11a-positive phagosome to form a complex with TRAM and TRIF, which then activates the TRAF3-TANK-binding kinase 1 (TBK1)-IRF3 axis to induce expression of type I IFN [142, 143] (Figure 1).

*(3) TLR5*. TLR5 is expressed on the cell surface and recognizes bacterial flagella [143]. Numerous studies have investigated the importance of MyD88-dependent TLR5 in pathogen phagocytosis and neutrophil/AMs/DCs clearance in the lung [144–148]. The antibacterial effect of intranasal flagellin in mouse model of *pneumococcus* respiratory infection indicates that flagellin has potential value as a therapeutic agent for the control of pulmonary infection [149]. In rodents, TLR5 is critical for mucosal intrinsic defense activity of *Salmonella enterica*, *P. aeruginosa*, *S. pneumoniae*, and uropathogenic *E. coli* [150, 151]. After bacterial recognition, plasma membrane-bound TLR5 recruits MyD88, followed by activation of NF-*κ*B through IRAKs, TRAF6, TAK1, and I*κ*B kinase (IKK) complexes to induce expression of inflammatory cytokines and chemokine gene, such as IL-8, IL-6, TNF-*α*, CXCL1, CXCL2, and CCL20 [152–155] (Figure 1).

#### 3.1.2. Endosome Membrane-Bound TLRs

*(1) TLR9*. Endosome membrane-bound TLR9 plays an important role in activating innate immunity by recognizing CpG-specific motifs found in microbial DNA [14]. For example, deficient in either TLR9 or MyD88 was impaired in bacteria uptake and clearing the airway infection caused by *S. pneumoniae* [156]. Mice lacking TLR9 are unable to produce Th1 effector cells, resulting in increased bacterial load in the lungs [130]. Thus, TLR9 plays a detrimental role in *P. aeruginosa* pneumonia and methicillin-resistant *S. aureus* pneumonia [157]. Additionally, a study indicated that *P. aeruginosa* DNA reduced IL-1*β* and NO production by TLR9 signaling, impairing the ability of activated AMs to clear bacteria [158]. Finally, after bacterial recognition, TLR9 activates MyD88-TRAF6-dependent NF-*κ*B to induce production of IFN and inflammatory cytokines/chemokines [15] (Figure 1).

### 3.2. NOD-Like Receptors and Inflammasomes in Pulmonary Infections

#### 3.2.1. NOD1 and NOD2

CARD mainly includes NOD1 and NOD2. Although NOD1 and NOD2 are similar in structure, their activated ligands have different roles in lung immunity. For example, compared with NOD2, NOD1 does not play an important role in immune response to *A. baumannii* infection [159, 160]. NOD1 recognizes *C. pneumoniae*, *L. pneumophila*, *K. pneumoniae*, *H. influenzae*, and *P. aeruginosa* [159, 161–163]. NOD2 plays an important role in host against viral and bacterial pathogens of the lung, such as *S. pneumoniae*, *S. aureus*, *E. coli*, *C. pneumoniae*, *M. tuberculosis*, and *A. baumannii* [159, 164].

**Figure 2:**
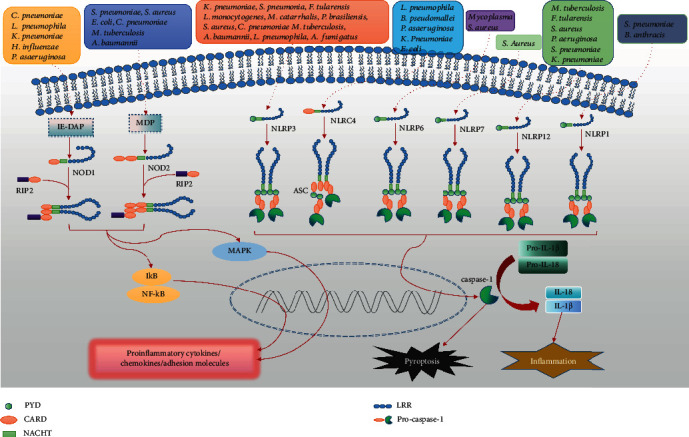
Overview of NLRs and RIG-I involved in pulmonary infections. Various lung pathogens are sensed by different NLRs. Cytosolic NOD1 and NOD2 recognize bacterial peptidoglycans (iE-DAP and MDP). After pulmonary pathogen activation, Nod1 and Nod2 activate downstream NF-*κ*B and MAPK through recruiting the serine-threonine kinase RIP2, leading to the production of proinflammatory cytokines, chemokines, and adhesion molecules. When stimulated by pulmonary bacterial pathogens, those molecules oligomerize to form the inflammasome, consisting of NLRP, ASC, and pro-caspase-1, which in turn cleaves pro-caspase 1 into its active form. Active caspase-1 can than cleaves the pro forms of IL-1*β* and IL-18 into their respective mature cytokines leading to inflammation and/or initiate pyroptosis. NOD: nucleotide-binding and oligomerization domain; NLR: nucleotide-associated oligomerization domain-like receptor; NLRP: NOD-like receptor proteins; iE-DAP: *γ*-D-Glu-mDAP; NLRC4: NOD-, LRR,- and CARD domain-containing-4; MD: MurNAc-L-Ala-D-isoGln; NF-*κ*B: nuclear factor-*κ*B; MAPKs: mitogen-activated protein kinases; ssRNA: single-stranded RNA; ASC: apoptosis-associated speck-like protein; CARD: caspase recruitment domain; PYD: pyrin domain; RIP2: receptor interacting protein 2; LRR: leucine-rich repeat; *C. pneumonia*: *Cryptococcus pneumoniae*; *B. pseudomallei*: *Burkholderia pseudomallei*; *C. neoformans*: *Cryptococcus neoformans*; *F. tularensis*: *Francisella tularensis*; *K. pneumoniae*: *Klebsiella pneumoniae*; *M. catarrhalis*: *Moraxella catarrhalis*; *P. brasiliensis*: *Paracoccidioides brasiliensis*; *Bacillus anthracis*: *B. anthracis*.

Previous studies characterizing NOD1 and NOD2 members of the NLR family have shown that these NLR have protective effects against microbial pathogens, and the lack of these NLR has led to increased morbidity and mortality in infected animals [165]. NOD1 and NOD2 in macrophages infected by *M. pneumoniae* coordinate host immune defenses by producing IFN-*γ*, nitrogen oxides, IL-12p40, and macrophage inflammatory protein-2 [135]. NOD1 and NOD2 activate NF-*κ*B and MAPK through the serine-threonine kinase RIP2, resulting in the production of proinflammatory cytokines, chemokines, and adhesion molecules (Figure 2).

#### 3.2.2. Inflammasomes

*(1) NLRP3*. The best characterized inflammasome is NLRP3, which is primarily up-regulated in immune and inflammatory cells after infection of multiple pathogens, such as *K. pneumoniae*, *S. pneumoniae*, *S. aureus*, *C. pneumoniae*, *C. neoformans*, *M. tuberculosis*, *L. pneumophila*, *Francisella tularensis*, *Moraxella catarrhalis*, *Listeria monocytogenes*, and *Paracoccidioides brasiliensis*, *A. fumigatus*, and *A. baumannii* [20, 158, 166–169]. The formation of the inflammasome leads to caspase-1 activation that triggers pyroptosis and activation of interleukin-1*β* (IL-1*β*) and IL-18 that eventually contributes to bacterial clearance [170, 171] (Figure 2). In recent years, the important protective role of the NLRP3 inflammasome is indicated by enhanced bacterial growth in the lungs of NLRP3 knockout (KO) and Asc knockout (KO) mice infected with serotype 2 *S. pneumoniae* (D39) [169]. Importantly, infected NLRP3 deficient (Nlrp3^−/−^) C57BL/6 mice failed to process and secrete IL-1*β* and displayed diminished bacterial clearance and incomplete innate immune cell activation compared to wild-type (WT) mice [172]. Those findings demonstrate that bacterial-infected mice activate NLRP3 inflammasome to produce inflammatory cytokines such as IL-1*β* [172–175]. In stark contrast to the current paradigm, infecting with lethal *S. pneumoniae* at increasing doses, results in NLRP3 inflammasome strongly impair host defense [169, 176, 177]. However, the current results are only reminded a detrimental role of ASC and NLRP3 in antibacterial defense during community-acquired pneumonia; it is still unclear that weather those downstream consequences of NLRP3 inflammasome activation is similar to those in the model of acute bacterial infection.

*(2) NLRC4*. Several lines of evidence show that NLRC4 recruits and activates caspase-1, which assembles inflammasome, leading to the pyroptosis and secretion of IL-1*β* and IL-18, thereby inhibiting *L. pneumophila* replication in mouse macrophages [178] (Figure 2). Additionally, NLRC4 is up-regulated in immune and inflammatory cells after infection of diverse bacteria, such as *Salmonella typhimurium*, *K. pneumoniae*, *L. pneumophila*, *P. aeruginosa*, *Burkholderia pseudomallei*, and *E. coli* [158, 179] (Figure 2). Besides, NLRC4 inhibits IL-17A-dependent neutrophil accumulation by inducing necroptosis and IL-18 activation in the lungs following *S. aureus* infections [180]. Thus, a novel therapeutic approach may be provided by modulating the function of the NLRC4 for the treatment of bacteria-induced infections in the lungs in the future.

*(3) NLRP6 and NLRP12*. It has been well established that NLRP6 regulates host defense inflammasome in response to bacterial infections. NLRP6 colocalizes with ASC, caspase-1, and caspase-11 forming the NRLP6 inflammasome complex to induce cell pyroptosis [165]. Once activated, pro-IL-1*β* and pro-IL-18 are converted into their active and proinflammatory forms, IL-1*β* and IL-18, ready for mediating further immunological responses [181, 182] (Figure 2). In contrast to the believed proinflammatory role, as other NLRs, NLRP6 is a negative regulator of inflammatory signaling to dampen host responses against several bacterial pathogens, thereby promoting bacterial dissemination [165]. For example, compared to their WT counterparts, NLRP6^−/−^ mice were highly resistant to infection with the pulmonary *S. aureus*, which were evidenced by improving survival rates and enhancing bacterial clearance in the lungs [183].

Similar to NLRP6, NLRP12 is another example of NLR family member that negatively regulates inflammatory response [184]. NLRP12 plays a protective role in *Yersinia pestis* infection by inducing IL-18-mediated IFN-*γ* secretion [185]. NLRP12^−/−^ mice had reduced survival rate and enhanced bacterial burden response to pathogenic bacteria infection, such as *M. tuberculosis*, *F. tularensis*, *S. aureus*, *P. aeruginosa*, *S. pneumoniae*, and *K. pneumoniae*, suggesting that NLRP12 has functional redundancy with other NLRs or has small contribution to lung inflammation [20, 158, 184, 186] (Figure 2). In conclusion, the disadvantageous roles of NLRP6 and NLRP12 have been depicted in innate immunity of the lungs. Blocking NLRP6 and NLRP12 respectively will augment immune-associated bacterial clearance, which should be considered as a potential therapeutic intervention strategy for attenuating the tissue injury induced by pulmonary infectious diseases.

*(4) Other Inflammasomes*. As with other inflammasome-forming NLR family members, such as NLRP3, the formations of the NLRP1 and NLRP7 inflammasomes lead to caspase-1 activation that trigger the pyroptosis and activation of IL-1*β* and IL-18, which eventually contribute to bacterial clearance [187–190] (Figure 2). NLRP7 responds to bacterial lipopeptides, Mycoplasma, and *S. aureus* infection by forming inflammasome [20, 190]. The NOD-, LRR-, and CARD domain-containing-5 (NLRC5) inflammasome is an important regulator of major histocompatibility complex I (MHC I) expression [191]. It was reported that NLRC5 is one of the largest members of the NLR family. However, the role of NLRC5 is unclear in pulmonary immune cells and immune-related tissues of inflammatory response case [192, 193]. It should be noted that the NLRC4 inflammasome can be activated by a wide range of pathogens or host-derived factors such as lipopolysaccharide (LPS) [22]. Along the same lines, it is possible that a wide range of pathogens and/or their components or host-derived ligands can activate NLRP5. However, the function of NLRC5 is redundant with inflammasome receptors NLRP3 and NLRC4 in host defense against *Salmonella* [194]. NLRC5 deficiency did not affect IL-1*β* production in response to various stimulations, including LPS and *F. tularensis* [194]. Indeed, the role of NLRC5 in pulmonary bacterial infection is extremely poor and remains to be improved. Likewise, the roles of the other members of the NLR family are still unclear in the lungs. A future goal is to explore the mechanisms of NLR and apply current understanding of NLR to reduce excessive inflammation while enhancing host defense during respiratory infections.

## 4. Conclusions

The lung contains a complex system of defense mechanisms during microbial infections. To protect the lung from microbes, the immune system forms several lines of defense. The first line of defense is established by innate immune cells (e.g., AMs, DCs, neutrophils, and NK cells). In addition, successful recognition and appropriate response of invading pathogens in the lung is essential for effective lung host defense. Recognition of cells can stimulate autophagy, phagocytosis, and clearance of necrotic cells and pathogens, further affecting local inflammatory responses. However, due to the complexity of the mechanisms, we still have a very limited perspective about the innate immune recognition of microbial pathogens and the interactions of different PRRs. Therefore, increased understanding of the PRR signaling recognition mechanisms during pulmonary pathogen infections will facilitate our knowledge of immune pathogenesis and lay the foundation for developing effective therapeutic measures.
